# Identification of biomechanical alterations associated with nonunion after tibial shaft fractures using musculoskeletal simulation based on motion capture and instrumented insole data

**DOI:** 10.1007/s00402-026-06518-7

**Published:** 2026-09-22

**Authors:** Tabea Wahl, Bergita Ganse, Elke Warmerdam, Frank Hildebrand, Ulf Krister Hofmann, Maximilian Praster

**Affiliations:** 1 Department of Orthopaedics, Trauma and Reconstructive Surgery, Universitätsklinikum Aachen, Aachen, Germany; 2 Werner Siemens-Endowed Chair for Innovative Implant Development (Fracture Healing), Departments and Institutes of Surgery, Saarland University, Saarbrücken, Germany

**Keywords:** Fracture healing, Weight bearing, Lower limb biomechanics, Gait asymmetry

## Abstract

**Introduction:**

Musculoskeletal simulations based on gait analysis data provide access to parameters such as joint reaction forces, muscle forces, and push-off moments, which cannot directly be measured in clinical practice. Applying these methods may help evaluate weight-bearing patterns and identify mechanical risk factors associated with the development of nonunion. This study aimed to investigate differences in the longitudinal development of forces in the musculoskeletal system between cases with and without union in tibial shaft fractures.

**Materials and methods:**

Longitudinal 3D marker-based motion capture and instrumented insole data of thirteen patients (8 union; 5 nonunion) with tibial shaft fractures were used to generate individualized musculoskeletal models using the AnyBody™ software.

**Results:**

In a linear mixed-effects model, the fractured limb showed impaired biomechanical parameters compared with the contralateral limb, including knee joint reaction forces (KJRFs), muscle forces, and push-off moments (all *p* < 0.001). During follow-up, parameters of the fractured leg increased over time, including KJRFs (*p* = 0.011), muscle forces (*p* ≤ 0.030), and ground reaction force (*p* = 0.008). The union group exhibited greater muscle forces, particularly in the soleus (*p* = 0.014) and gastrocnemius muscles (*p* = 0.049). The nonunion group exhibited significantly greater asymmetry between healthy and fractured leg than the union group in ground reaction force (β = 0.368, *p* = 0.011), KJRFs (β = 0.364, *p* = 0.028), and push-off moment (β = 0.457, *p* = 0.028). In additional time point–specific analyses no significant differences were found at 6 and 12 weeks.

**Conclusion:**

Biomechanical loading patterns differed between patients with union and nonunion throughout fracture healing, with greater between-limb asymmetries in push-off moment, joint reaction forces, and plantar flexor muscle forces in the nonunion group. Larger studies are required to validate these findings using the proposed workflow.

## Introduction

Nonunion of long bones is a well-recognised and common complication, and as such it has been the subject of extensive research aimed at identifying associated risk factors. In cases involving high-energy trauma, bone loss, or patient-related factors such as smoking, type 2 diabetes mellitus, alcoholism, or elevated body mass index, the risk of nonunion is increased [1–3]. The precise time point at which a fracture should be classified as nonunion remains the subject of debate [4]. However, a general consensus exists that a healing period exceeding six months should be considered a nonunion [5, 6]. Reported malunion and nonunion rates following tibial fractures vary widely across studies, ranging from 5.4% [7] and 6.8% [3] up to 12% [8] and 13.3% [9]. Furthermore, patients are notably young with a peak incidence between 30 and 44 years [7]. Predictable risk factors for nonunion in tibial shaft fractures include open fractures, high-energy trauma, and compromised soft tissue conditions [3, 8, 10, 11]. In cases of malunion or nonunion, patients not only require significantly longer periods to return to work and report prolonged pain [12], but also experience restricted mobility and limb shortening [13]. In addition, the nonunion status has been demonstrated to be associated with increased and prolonged opioid consumption, as well as nearly doubled healthcare costs [8].

Fracture healing requires a delicate balance between stability and mechanical stimulation, depending on fracture characteristics and the choice of surgical technique. Absolute stability, typically achieved through rigid fixation methods, minimalizes interfragmentary motion and promotes primary bone healing. In contrast, relative stability, commonly provided by intramedullary nailing, allows controlled micromotion at the fracture site, which stimulates secondary bone healing through callus formation [14, 15]. Tibial shaft fractures are commonly treated with intramedullary nailing, as its load-sharing characteristics enable early postoperative weight-bearing while maintaining sufficient stability for successful healing [16].

In daily clinical practice, weight-bearing recommendations are based on a limited amount of scientific evidence and are often difficult to implement due to poor patient compliance [17, 18]. However a number of studies have indicated a potential correlation between early weight bearing, shorter time to bone union, and reduced nonunion rates in tibial fractures, suggesting that weight-bearing behaviour may represent a clinically relevant parameter for assessing fracture consolidation [16, 19]. Conversely, excessive loading at an early stage may lead to pronounced callus formation but delayed fracture healing [20].

Clinical assessment tools such as gait analysis are gaining increasing attention due to their potential as non-invasive and radiation-free methods for evaluating the healing process of fractured bones in the lower limb [21]. Gait analysis gives clinicians an impression of the functional progress during healing by analysing ground reaction forces, while also providing valuable information regarding parameters such as limb asymmetry, range of motion, gait speed and step length [22].

In the field of lower leg fracture research, several studies have demonstrated that a range of gait parameters can serve as valuable tools for assessing functional progress in fracture healing. Six months after a fracture of the tibial shaft, significant deficits in parameters such as knee flexion during the loading response and ankle dorsiflexion during the stance phase remain evident compared to the contralateral, uninjured leg [23]. Larsen et al. reported an increase in gait speed and cadence between the six- and twelve-month follow-ups, accompanied by a reduction in single support phase asymmetry from 12.8% at six months to 3.8% at twelve months, as well as a decrease in step length asymmetry (11.9% at six months and 5.1% at twelve months) [24]. Comparing healed fractures with nonunified fractures, Warmerdam et al. demonstrated that the nonunion group exhibited significantly shorter stance times both six weeks and six months after surgery [25]. The findings of this study indicate early alterations in gait patterns, which may allow for the identification of potential risks for the development of nonunion at an early stage.

Musculoskeletal simulations provide additional opportunities to extend conventional gait analysis by enabling the estimation of parameters such as joint reaction forces, muscle forces, and joint moments. These parameters may contribute to a more comprehensive understanding of the biomechanical factors relevant to the early detection of delayed fracture healing or nonunion. Recent studies investigating differences in loading between union and nonunion cases have either focused on gait parameters or incorporated musculoskeletal simulations primarily as boundary conditions for finite element models [26, 27]. However, the forces calculated directly by the musculoskeletal simulations themselves have not yet been systematically analysed or evaluated regarding their potential as tools for assessing fracture healing.

Therefore, the primary aim of this study was to investigate longitudinal differences in musculoskeletal-derived biomechanical parameters between union and nonunion cases following tibial shaft fractures. We aimed to identify alterations in weight-bearing–related parameters during fracture healing that may be associated with impaired healing outcomes. Furthermore, this study explored whether simulation-derived parameters, including joint reaction forces, muscle forces, and push-off moments, could provide additional insights into gait adaptations and serve as potential predictors of nonunion.

## Materials and methods

The patient data used in this study were provided by the Department of Innovative Implant Development (Fracture Healing) at Saarland University, Germany, based on a collaboration agreement. The data collection period ranged from June 2022 to April 2024.

From the overall study cohort, only patients with tibial shaft fractures were included in the present analysis. Exclusion criteria were age younger than 18 years, inability to provide informed consent, pregnancy, presence of concomitant injuries or conditions that impaired mobility. The patients were grouped into a union and a nonunion group. Nonunion was defined as the absence of bridging callus on radiographs obtained 6 months after surgery, based on assessment by an experienced orthopaedic surgeon. For further details on the study design, see Warmerdam et al.

### Measurements

**Fig. 1:**
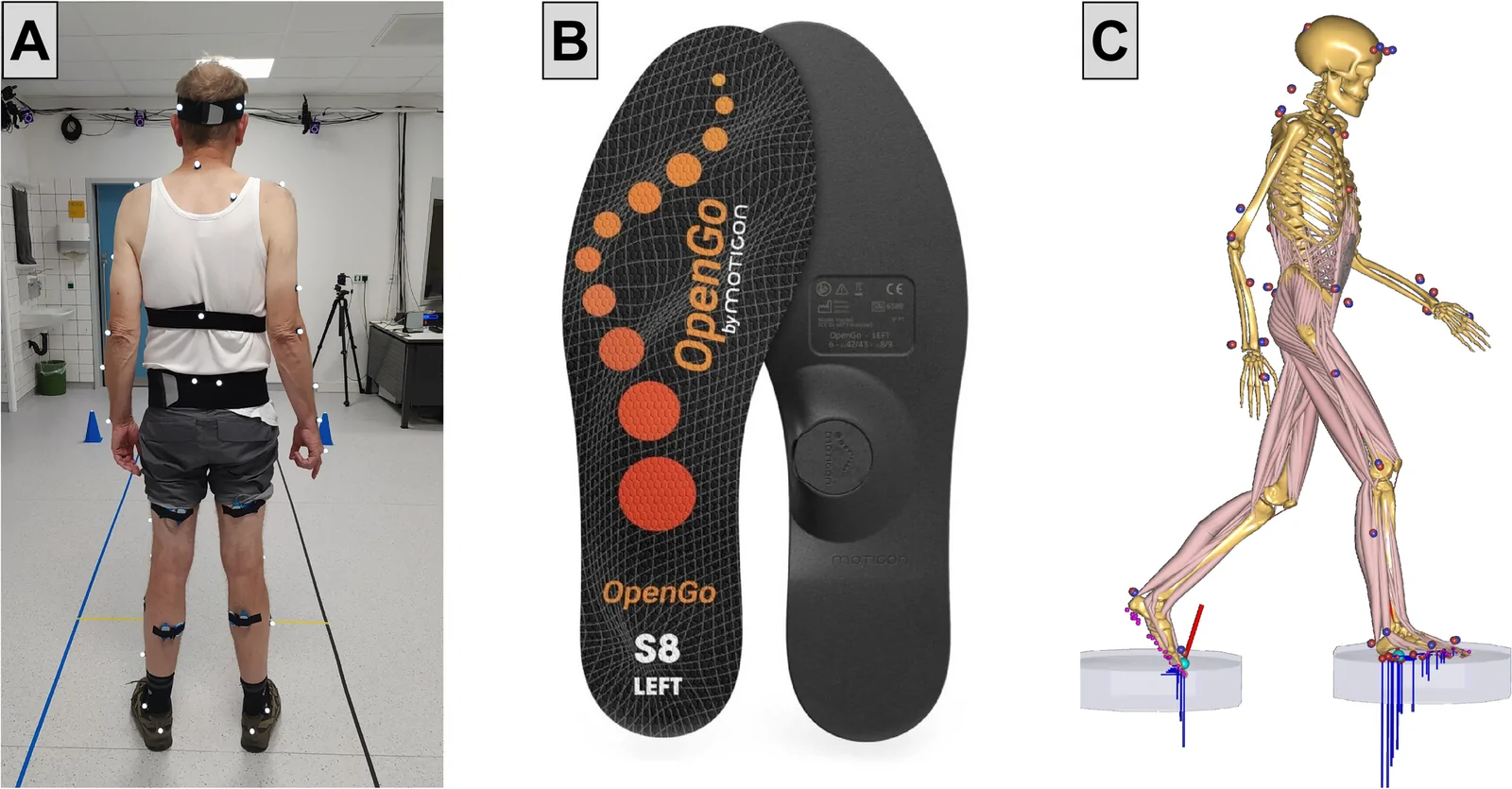
Experimental setup and musculoskeletal modeling workflow **A** Healthy subject wearing reflective markers for motion capture in the gait laboratory. **B** Moticon OpenGo instrumented insoles used to measure vertical ground reaction forces. **C** Musculoskeletal simulation in the AnyBody Modeling System illustrating the digital twin with marker trajectories and ground reaction forces

The patients underwent surgical intervention at Saarland University Hospital. Postoperatively, measurements were conducted in a gait laboratory on multiple occasions to capture longitudinal changes in gait patterns. Measurements were obtained as early as 8 days postoperatively and up to one year after surgery. The median follow-up duration was 189 days (range: 42–361 days). During the early stages of the follow-up period, patients were permitted to ambulate with the assistance of crutches with a weight-bearing restriction of 20 kg. In some cases, a transition phase was recommended, characterized by the adaptation of weight bearing activities to the patient’s pain level. Following this adjustment, patients were permitted to bear full weight. Gait analysis was performed using a 13-camera optical motion capture system (Vicon Vero, Oxford Metrics, UK). To enable full-body motion capture, up to 40 reflective markers were placed on each patient according to the Vicon Plug-in Gait Model (Fig. 1). Patients were instructed to walk a 10-meter distance at a comfortable, self-selected speed, in accordance with their prescribed weight-bearing restriction, and using crutches when required. The central 6-meter section of the walkway was analyzed to ensure steady state walking. Simultaneously, plantar pressure data were recorded using wireless pressure-sensing insoles (Moticon OpenGo, Moticon ReGo AG, Munich, Germany), which the patients wore in their own sports-shoe style footwear. The system consists of 16 plantar pressure sensors, which record the vertical ground reaction force (vGRF) at a sampling frequency of 100 Hz (Fig. 1).

### Musculoskeletal simulation

Musculoskeletal modeling was performed using the AnyBody Modeling System 8.1 (AnyBody Technology, Aalborg, Denmark), a software platform widely applied in biomechanics, ergonomics, sports science, and medical research. For each trial, an individual digital twin was generated, resulting in a total of 35 subject-specific AnyBody models. The scaling of the model was based on the individual patient´s height and body mass to ensure physiologically accurate force calculations. All simulations were embedded in the Full Body GRF Prediction Model from the AnyBody Managed Model Repository (AMMR). The motion capture data, acquired with the Vicon system, were imported into the AnyBody software as .c3d (Coordinate 3D) files. Marker configurations were adapted to the experimental setup, and parameter identification was performed for each trial. To incorporate the vertically measured GRF obtained from the pressure insoles, a custom add-on code was developed and integrated into the Full Body GRF Prediction framework. The measured vGRF was applied at the center of pressure (CoP) to a virtual force plate rigidly attached to the foot segment. The force plate estimated horizontal GRF components in the anterior–posterior and mediolateral directions based on the applied vertical force. This approach enabled the estimation of frictional forces and horizontal loading, which are necessary for the realistic computation of joint reaction forces, without requiring force plates. Afterwards, inverse dynamics analysis was conducted. Parameters exported from the AnyBody Modeling System and used for statistical analysis included joint reaction forces of the lower limb (hip, knee, and ankle), muscle forces of the plantar flexors (soleus and gastrocnemius), the ankle plantarflexion moment (push-off moment), and ground reaction forces. The push-off moment is primarily generated by the triceps surae, additional foot muscles, and the elastic recoil of the Achilles tendon [28, 29], producing a positive power peak that is important for increasing gait speed through forward acceleration of the body mass and improved energy efficiency [30, 31]. The resultant joint reaction forces (JRF) were calculated using the following equation:

$$ JRF = \sqrt {proximodistalJRF^{2} + mediolateralJRF^{2} + anteroposteriorJRF^{2} } $$

### Statistical analysis

Statistical analyses were performed using MATLAB R2022b (MathWorks, Natick, MA, USA). The statistical significance was set at α = 0.05 and all statistical tests were two-sided. To account for multiple comparisons across outcome variables, p-values were adjusted using the Benjamini–Hochberg procedure to control the false discovery rate (FDR). This approach was chosen to balance the risk of type I errors with statistical power in this exploratory study with a limited sample size.

For each step, peak values of the respective parameters were identified. Subsequently, mean values were calculated for each limb within a trial based on all included steps.

To quantify functional differences between the fractured and contralateral limbs, an asymmetry index (AI) was calculated for all musculoskeletal variables. The AI represents a normalized, dimensionless measure of inter-limb differences and was defined as:

$$ AsymmetryIndex = \frac{{HealthyLeg - InjuredLeg}}{{0.5x(HealthyLeg + InjuredLeg)}} $$

Here, *Healthy Leg* and *Injured Leg* denote the mean values of the respective parameter for the contralateral and fractured limb, respectively. An AI value of 0 indicates perfect symmetry, while positive and negative values indicate higher values in the healthy and injured limb.

This formulation is consistent with commonly used symmetry indices based on normalized inter-limb differences [32].

#### Linear mixed-effect models

To investigate the relationship between musculoskeletal variables, trial progression, and clinical outcome, linear mixed-effects models (LMMs) were applied. LMMs were selected because the dataset contained repeated measurements from the same participants across multiple trial days. In the present study, musculoskeletal outcome variables (e.g., joint moments, muscle forces, and push-off moment) served as dependent variables. Trial day and outcome group were included as fixed effects to evaluate temporal changes and potential group differences. Initially, an interaction term between trial day and outcome was included to assess whether temporal changes differed between groups. If the interaction terms were not found to be statistically significant, the final models were refitted without the interaction term. To account for inter-individual variability and the repeated-measures structure of the data, participant-specific random intercepts were included in the models. The final model structure was defined as:

$$ {\text{Dependent Variable}}\sim {\mathrm{TrialDay}} + {\mathrm{Outcome}} + \left( {{\mathrm{1}}|{\mathrm{Patient}}} \right) $$

TrialDay was treated as a continuous variable representing time since surgery, and outcome denoted group classification. Model parameters are reported as regression coefficients (β), representing the expected change in the dependent variable per unit increase in the predictor. Specifically, β for TrialDay indicates the change per day, while β for Outcome reflects the difference between groups (nonunion relative to union or healthy relative to fractured limb). Models were estimated using maximum likelihood estimation. Model assumptions were assessed by visual inspection of residuals using Q–Q plots and histograms. Residuals were approximately normally distributed, and no major deviations from model assumptions were observed. The statistical significance of model effects was assessed using F-tests and t-tests derived from the mixed-effects modeling framework.

#### Mann-Whitney U test

The Mann–Whitney U test was applied to identify early differences in musculoskeletal parameters at fixed time points. Due to the small sample size and deviations from normality, this non-parametric test was chosen. To ensure that all patients have parameters at the same time, a linear interpolation was employed to calculate the values. The selected time points for the test were 42 days (6 weeks) and 84 days (12 weeks). Parameters used for this test were the Asymmetry Indices of all values and the Benjamini-Hochberg FDR procedure was also applied to this test.

## Results

### Patient characteristics of the union and nonunion group

Thirteen patients with tibial shaft fractures were included in this study (Table 1). In eleven cases, definitive fracture fixation was performed using an intramedullary tibial nail (Stryker T2 Tibia), which allows immediate weight bearing according to AO recommendations. The remaining two patients underwent a plating procedure, primarily due to the distal location of the fracture. In case of one patient, radiographic data were not available in the hospital system. It was observed that all thirteen patients sustained an additional fibular fracture, which was treated with a plate fixation (*n* = 4), intramedullary nailing (*n* = 5) or conservative treatment (*n* = 4). Initially, seven patients received temporary stabilisation of the tibia with an external fixator and underwent definitive intramedullary nailing after several days. Among the thirteen patients, five developed tibial nonunion and did not achieve fracture union during the observation period (median follow-up: 214 days; range: 189–251 days). One additional patient developed nonunion of the fibula only. In eight cases, fracture union of the tibia occurred within the expected time frame of six months. One patient with nonunion and plate fixation underwent revision surgery four months after treatment due to broken screws. The procedure was changed to intramedullary nailing. Of the patients with tibial nonunion, one presented with arterial hypertension and diabetes mellitus, while another patient suffered from diabetes mellitus and was an active smoker.

**Table 1:** Patients characteristics of union and nonunion group of tibial shaft fractures

Variable	Union (*n* = 8)	Nonunion (*n* = 5)	*p*-value
Sex (Male/Female)	6 / 2	4 / 1	1.00
Age (years)	49.6 ± 17.7	51.0 ± 23.9	0.91
Body weight (kg)	86.3 ± 16.9	88.8 ± 9.0	0.73
Body height (cm)	178.1 ± 9.6	178.8 ± 4.4	0.87
Patients smoking	0	1	0.38
Patients with diabetes	0	2	0.13
Patients with hypertension	0	1	0.38

### Reduced musculoskeletal loading in the nonunion group in simulation analyses

**Fig. 2:**
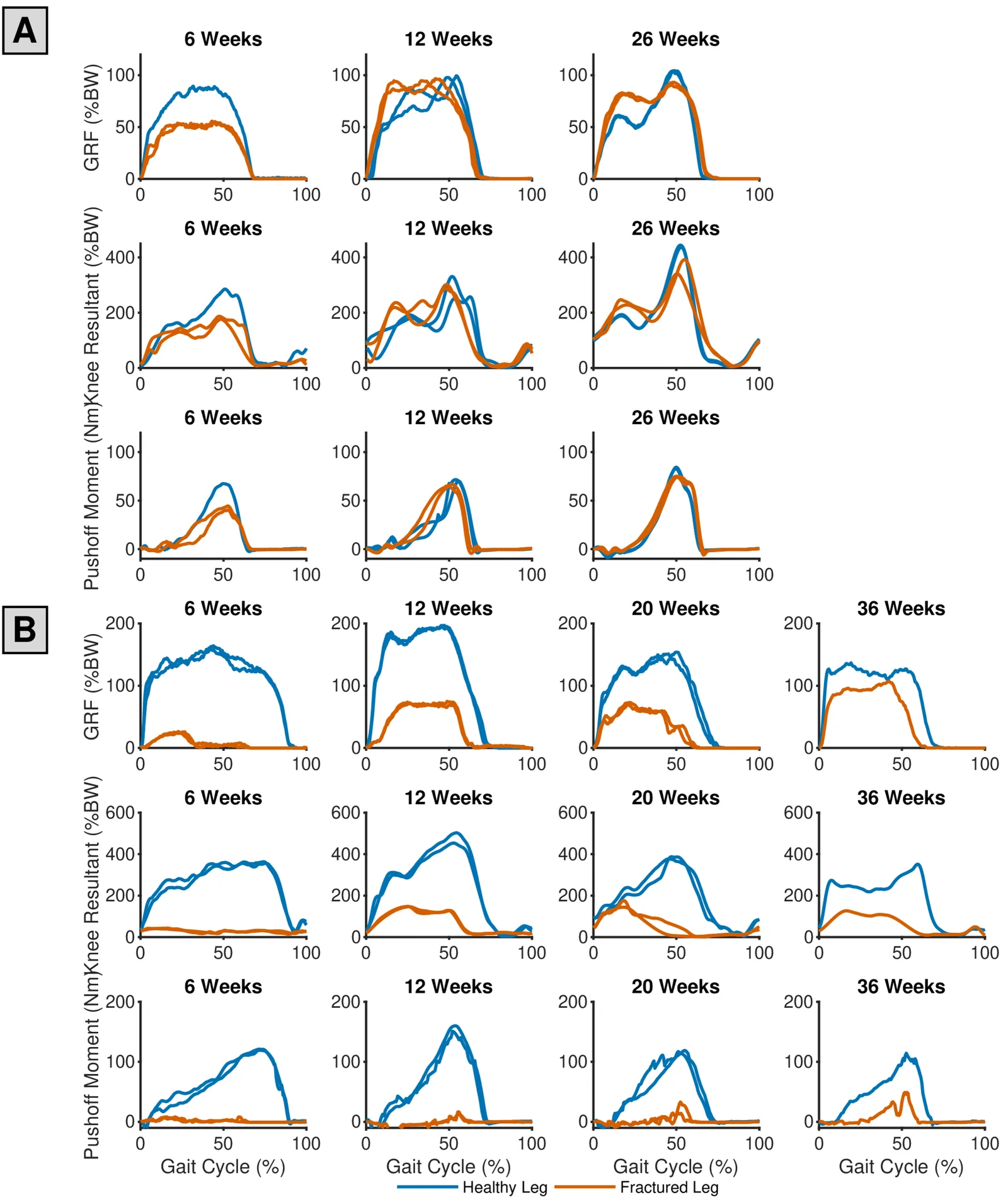
Ground reaction force, knee joint reaction forces, and push-off moments across the gait cycle for two representative patients after tibial shaft fracture. Blue lines represent the healthy limb, and red lines represent the fractured limb. **A** Patient 1 is a 64-year-old male who sustained a spiral fracture of the tibial shaft, accompanied by a proximal fibular fracture. Fracture healing occurred within the expected time frame of six months. **B** Patient 2 is a 79-year-old male with an intact wedge fracture, a history of diabetes mellitus, and active smoking, who subsequently developed a nonunion. Even after more than ten months, radiographic imaging failed to demonstrate adequate callus formation or bridging of the fracture gap. *%BW* percent body weight

All patients underwent gait analysis measurements over a follow-up period that extended up to one year. Three patients only participated in a single gait analysis, whereas others contributed up to five measurements. The longitudinal development of two representative patients is illustrated in Fig. 2. Although the patient with union showed generally lower GRFs, the fractured leg already showed forces as high as the healthy leg after 12 weeks, and the gait pattern shows a transformation to what is called a physiological gait, consisting of a double-peak. The first peak occurs during the loading response at heel contact, and the second during push-off [33]. Knee joint reaction forces (KJRFs) in the patient with a united fracture returned to a more physiological pattern after 26 weeks. In contrast, the patient with a non-united fracture exhibited GRFs that did not reach the level of the healthy limb, even after 36 weeks, with KJRFs remaining visibly lower. Notably, differences between the two patients were already observable six weeks post-surgery.

### Statistical results

#### Reduced loading of the fractured limb compared with the contralateral limb

Initially, an LMM was used to compare the healthy and fractured leg across all time points. All biomechanical parameters were found to be significantly lower in the fractured leg (Table 2). GRF was decreased (β = 256.17, *p* < 0.001) and similarly, JRF values were significantly lower in the fractured leg, including the knee (β = 130.31, *p* < 0.001), ankle (β = 270.41, *p* < 0.001), and hip (β = 108.07, *p* < 0.001). Muscle forces were also significantly lower in the fractured leg, including the soleus (β = 52.96, *p* < 0.001) and gastrocnemius (β = 88.45, *p* < 0.001). Furthermore, the push-off moment was found to be considerably lower (β = 47.26, *p* < 0.001).

#### Greater interlimb asymmetries in patients with nonunion compared with union

For the analysis of absolute values, only the fractured leg was considered. The LMM incorporating the TrialDay × Outcome interaction did not reveal significant interaction effects for most parameters. Since the interaction term was not significant and did not improve model fit, it was removed, and models were refitted including only the main effects.

When comparing union and nonunion cases over the observation period, several biomechanical parameters of the fractured leg increased significantly, indicating progressive recovery over time (Table 3). GRF (β = 1.530/day, *p* = 0.008), KJRFs (β = 0.651/day, *p* = 0.011), and hip joint reaction forces (β = 0.713/day, *p* = 0.006) increased considerably during follow-up. When comparing outcome groups, patients with union exhibited greater muscle forces compared to nonunion patients (Table 3). Plantar flexor muscle forces were significantly greater in the union group, including the soleus (β = −43.813, *p* = 0.014) and gastrocnemius (β = −68.186, *p* = 0.049).

The analysis of the asymmetry index revealed a progressive reduction of gait asymmetry between the healthy and fractured leg over time (Table 3). Significant improvements were observed for several parameters, including KJRFs (*p* = 0.014), muscle forces (*p* ≤ 0.020) and GRF asymmetry (*p* = 0.008). Furthermore, patients in the nonunion group exhibited considerably higher asymmetry in comparison to the nonunion group across multiple variables, particularly KJRFs (β = 0.364, *p* = 0.028), gastrocnemius and soleus force (β = 0.542, *p* = 0.019, β = 0.747, *p* = 0.001), push-off moment (β = 0.457, *p* = 0.028) and GRF (β = 0.368, *p* = 0.011).

To assess group differences between union and nonunion at specific time points, the Mann–Whitney U test was applied (Table 4). For this test 3 patients needed to be excluded due to their participation in only one measurement (one patient with nonunion and two patients grouped as union). At 6 weeks (42 days), differences between groups were observed for several parameters in unadjusted analyses. However, none of these differences remained statistically significant after correction for multiple comparisons. At 12 weeks (84 days), no differences between the groups were detected for any of the analysed parameters.

**Table 2:** Results of the linear mixed-effects models comparing biomechanical variables between the fractured and healthy leg

Variable	Outcome (β, 95% CI)	*p*-value (FDR corrected)
Ground reaction force (N)	256.17 (163.77 to 348.57)	< 0.001
Knee reaction force (%BW)	130.31 (87.48 to 173.15)	< 0.001
Ankle reaction force (%BW)	270.41 (197.01 to 343.81)	< 0.001
Hip reaction force (%BW)	108.07 (61.56 to 154.58)	< 0.001
Soleus force (%BW)	52.96 (35.77 to 70.15)	< 0.001
Gastrocnemius force (%BW)	88.45 (56.54 to 120.35)	< 0.001
Push-off moment (Nm)	47.26 (32.66 to 61.86)	< 0.001

Values represent the estimated effect (β) with 95% confidence intervals, with patient included as a random intercept

*β* regression coefficient, *CI* confidence interval, *%BW* percent body weight

**Fig. 3:**
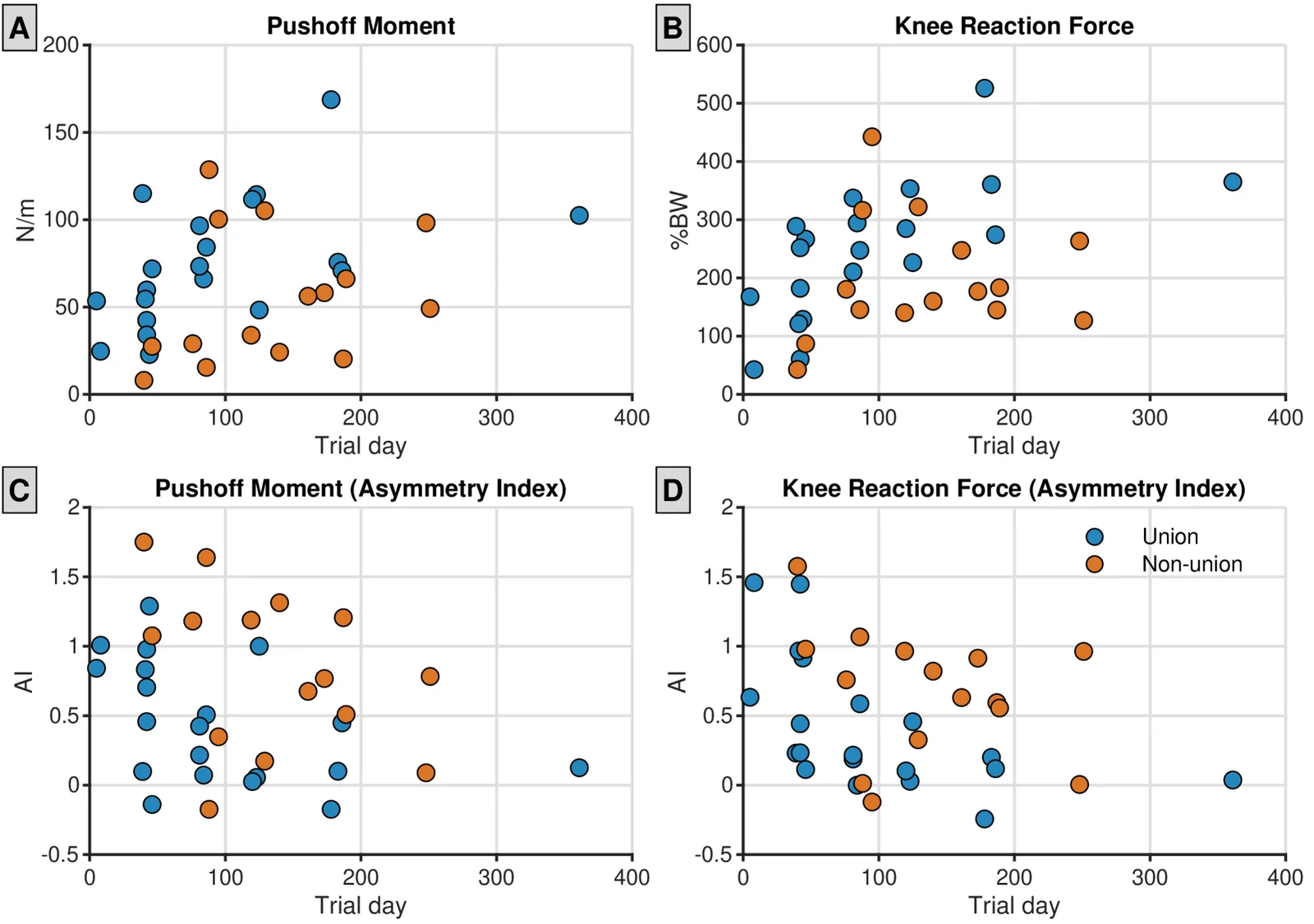
Relationship between trial day and ankle plantar flexion (Push-off) moment **A** and knee joint reaction force **B**, as well as the corresponding asymmetry indices **C**, **D**. Blue markers represent patients with fracture union, whereas orange markers represent patients with nonunion

**Table 3:** Linear mixed effects model of fractured leg values and asymmetry-index comparing union and nonunion

Outcome variable	Union vs. Nonunion			
	Mean values		Asymmetry Index	
	β TrialDay (*p*)	β Outcome (*p*)	β TrialDay (*p*)	β Outcome (*p*)
Ground reaction force (*N*)	**1.530 (0.008)**	-171.450 (0.071)	**-0.003 (0.008)**	**0.368 (0.011)**
Knee joint reaction force (%BW)	**0.651 (0.011)**	-76.506 (0.069)	**-0.003 (0.014)**	**0.364 (0.028)**
Ankle joint reaction force (%BW)	0.279 (0.490)	-69.613 (0.313)	-0.001 (0.590)	0.296 (0.316)
Hip joint reaction force (%BW)	**0.713 (0.006)**	-21.647 (0.632)	**-0.002 (0.014)**	0.240 (0.179)
Soleus muscle force (%BW)	**0.197 (0.030)**	**-43.813 (0.014)**	**-0.003 (0.020)**	**0.747 (0.001)**
Gastrocnemius muscle force (%BW)	**0.445 (0.011)**	**-68.186 (0.049)**	**-0.003 (0.014)**	**0.542 (0.019)**
Push-off moment (Nm)	**0.198 (0.013)**	-26.645 (0.071)	**-0.003 (0.014)**	**0.457 (0.028)**

*β* regression coefficient, *p * p-value (FDR-corrected), *GRF* ground reaction force, *KJRF* knee joint reaction force, *%BW * percent body weight

## Discussion

The aim of this study was to examine whether musculoskeletal simulation–derived biomechanical parameters differ between patients with union and nonunion following tibial shaft fractures. By analysing longitudinal changes in gait-related loading characteristics, including joint and muscle forces, we sought to evaluate their potential relevance as indicators of impaired fracture healing. During follow-up, gait mechanics progressively recovered, as reflected by increasing limb loading, joint reaction forces, muscle forces, and push-off moment. Nevertheless, the fractured limb remained underloaded, with lower GRF and push-off moment than the contralateral limb.

When comparing patients with union and nonunion, no significant interaction between time and outcome was observed, providing no clear evidence that the temporal progression of biomechanical parameters differed between the groups. However, the estimated overall group differences indicated lower plantar flexor muscle forces in patients with nonunion, amounting to 43.8%BW for the soleus and 68.2%BW for the gastrocnemius.

The asymmetry analysis supported these findings, showing greater between-limb asymmetry in patients with nonunion regarding lower ground reaction force, knee joint reaction forces, muscle forces, and push-off moment compared to patients with union (Fig. 3). In additional time point–specific analyses, group differences were observed at six weeks postoperatively in the unadjusted analyses, suggesting possible early alterations in gait mechanics in patients with nonunion. However, these differences did not remain statistically significant after correction for multiple comparisons, and no statistically significant differences were detected at twelve weeks. These findings should be interpreted considering the variable number and timing of follow-up measurements. Linear interpolation enabled comparisons at standardized postoperative time points but may not capture short-term or nonlinear changes. Therefore, interpolated values should be regarded as estimates.

Taken together, the findings suggest that fracture-healing outcome may be associated with postoperative loading patterns. The lower muscle forces, reduced push-off generation, and greater asymmetry observed in patients with nonunion may reflect a more protective loading strategy. However, the observational design does not allow the direction of this association to be determined: reduced loading may contribute to an unfavourable mechanical environment at the fracture site, but it may also result from pain, functional impairment, or an already unfavourable healing course. The observed tibial nonunion rate of approximately 38% was considerably higher than that generally expected in patients with tibial shaft fractures. Given the small sample size of only 13 patients, the observed nonunion rate is subject to considerable statistical uncertainty and should therefore not be interpreted as representative of the incidence of nonunion in the general population.

**Table 4:** Comparison of asymmetry indices for all biomechanical outcome variables between the union and nonunion group at 6- and 12-weeks using Mann–Whitney U tests values

Variable	Uncorrected *p*-value (42 days)	Uncorrected *p*-value (84 days)	Corrected *p*-value (42 days)	Corrected *p*-value (84 days)
Ground reaction force (N)	0.762	0.352	0.821	0.411
Knee joint reaction force (%BW)	**0.038**	0.171	0.133	0.300
Ankle joint reaction force (%BW)	0.067	1	0.187	1.000
Hip joint reaction force (%BW)	0.352	0.257	0.411	0.360
Soleus muscle force (%BW)	**0.001**	0.171	0.133	0.300
Gastrocnemius muscle force (%BW)	**0.038**	0.171	0.133	0.300
Push-off moment (Nm)	**0.038**	0.257	0.133	0.360

Bold values indicate statistically significant uncorrected *p*-values (*p* < 0.05); corrected *p*-values are provided to account for multiple comparisons

*%BW * percent body weight

Traditional treatment strategies for nonunion often aim to reduce interfragmentary strain through decreased weight bearing or increased fixation rigidity [34, 35].

In contrast, clinical evidence indicates that tibial shaft fractures treated with intramedullary nailing may benefit from early weight bearing [16, 19]. Experimental data from a canine model demonstrated that increased mechanical loading, induced by unloading the contralateral limb, resulted in enhanced blood flow, increased new bone formation, and improved mechanical properties, including rotational stability and energy absorption [36]. Numerous studies have further shown that axial compressive interfragmentary strain promotes fracture healing [37–40], whereas excessive shear movement delays healing and reduces periosteal callus formation [34]. Taken together, these findings highlight the complex and often contradictory role of mechanical loading in fracture healing.

In line with this, a previous study in the same cohort reported shorter, more asymmetric stance times in nonunion patients at six weeks and six months, and lower gait speed, step height, and knee ROM at three months [25]. We extended this subgroup by adding plantar pressure data to a musculoskeletal simulation, yielding joint and muscle forces and push-off moments not previously assessed. These simulation-derived asymmetry indices did not reach significance at 6 or 12 weeks after correction possibly reflecting differences in parameter type, power, or correction stringency rather than absent early alterations. Furthermore, another study demonstrated that as early as six weeks after a tibial shaft fracture, 16 out of 33 assessed gait parameters showed considerable reduction in the nonunion group, indicating that gait analysis may allow earlier detection of potential nonunion compared to radiographic imaging [27].

Joslin et al. reported similar observations in a cohort of twelve patients, where ten patients with successful fracture healing progressively increased weight bearing to approximately 90% of the uninjured limb before removal of the fixator, while the two patients with nonunion achieved only 40% loading at 20 weeks [41].

Gait analysis can already be a time-consuming tool, as it requires bringing the patient to the laboratory, applying markers, and repeatedly performing walking trials. Incorporating an additional step through musculoskeletal simulation further increases the required time. Computer models such as Anybody provide the opportunity to estimate forces that can otherwise only be measured directly through the implantation of instrumented devices during arthroplasty [42, 43]. Although the simulation-derived parameters did not demonstrate a clear advantage over conventional gait measures in this small cohort, the proposed workflow represents an important step towards a patient-specific assessment of the mechanical environment during fracture healing. Unlike conventional gait analysis and instrumented insoles, musculoskeletal simulations enable the estimation of internal forces that cannot be measured directly in vivo. These forces could subsequently be integrated into fracture-specific models to estimate force transmission, interfragmentary motion, and strain within the fracture gap. While the workflow is currently more time-consuming than conventional approaches, the development of automated, standardized, and patient-specific analysis pipelines could substantially reduce processing time and facilitate its future clinical translation. The present findings therefore provide a foundation for larger longitudinal studies investigating whether this approach offers additional diagnostic or prognostic value and may ultimately support individualized rehabilitation and weight-bearing recommendations.

It is important to note that fracture healing is influenced by a wide range of additional factors beyond mechanical loading. The diamond concept emphasises that, in addition to mechanical stability, biological factors such as osteogenic cells, osteoinductive mediators, and an osteoconductive scaffold are essential for successful bone regeneration [44]. Patient-specific risk factors, including diabetes mellitus, smoking, arterial hypertension, and obesity, further increase the risk of nonunion. Moreover, fracture characteristics, such as high-energy trauma and open fractures, are well-established predictors of impaired healing [10]. Differences in vertical ground reaction force may also be influenced by gait speed [45], which in turn increases loading conditions within the fracture gap [46].

Furthermore, all patients in the present study sustained both tibial and fibular fractures, a common clinical scenario, as approximately 58.2% of patients with tibial fractures present with at least one additional bony injury [47]. The presence and treatment of fibular fractures have been demonstrated to influence tibial fracture healing by altering load transmission. Orth et al. suggested that fibular fractures at the same level as the tibial fracture can influence force distribution. They further postulated that stabilising the fibula can reduce maximum strain within the tibial fracture gap, thereby supporting surgical fixation. In contrast, proximal fibular fractures appear to have a limited impact on tibial healing and may therefore be managed conservatively [46, 48].

After fractures of the lower leg, partial weight bearing is commonly prescribed using a fixed load and duration. However, this standardized approach does not account for individual differences between patients. Factors such as fracture type, comorbidities associated with an increased risk of nonunion, and the patient’s pain level may substantially influence the optimal mechanical loading during rehabilitation. A more individualized approach to weight-bearing prescriptions could therefore help determine a patient-specific loading regime that both maximizes the potential for fracture healing and improves patient compliance. Future clinical application of this approach could involve regular gait analysis during physiotherapy sessions, for example, at biweekly intervals, combined with individualized feedback on permitted weight bearing. The integration of assistive technologies, such as pressure sensing insoles or even smart crutches [49] capable of monitoring loading patterns, could further enable real-time feedback and improve patient compliance. In combination with musculoskeletal simulations, such approaches may ultimately allow the development of personalized, load-adapted rehabilitation strategies following tibial shaft fractures.

Although this study included a relatively small sample size, the observed differences in biomechanical parameters suggest that alterations in gait mechanics may be associated with the subsequent development of nonunion.

Lower plantar flexor muscle forces, reduced knee joint reaction forces, and lower ground reaction forces were observed in patients who subsequently developed nonunion, suggesting an association between reduced mechanical loading and impaired fracture healing. However, these findings should be considered exploratory and do not establish a predictive relationship.

### Limitations

This study has several limitations that should be considered when interpreting the results. The sample size was relatively small, and the number of gait trials per patient was limited, with some participants contributing only one or two measurements. Consequently, the statistical power of the analyses is limited, and the findings should be considered exploratory. Given the observational study design, no conclusions can be drawn regarding the direction of the observed association. Lower postoperative loading may represent a factor involved in impaired fracture healing, but it may equally be a consequence of an already unfavourable healing course accompanied by pain or functional limitations. The available data therefore do not allow the mechanisms underlying the differences in loading patterns between the groups to be identified. Also, due to the limited cohort size, it was not possible to perform subgroup analyses, for example, comparing different surgical techniques or stratifying patients according to fracture type. Future studies including larger patient cohorts, more frequent follow-up measurements, and complementary clinical assessments are therefore needed to further investigate the temporal and potentially causal relationship between postoperative loading patterns and fracture healing. Although PROMs (patient-related outcome measurements), such as patients’ pain, functional status, and health-related quality of life were collected as part of the broader study protocol, they were not included in the present analysis, which focused specifically on biomechanical loading patterns. Future studies should integrate both measures to provide a more comprehensive assessment of recovery. Additionally, potential confounding factors such as differences in rehabilitation protocols, patient compliance, and gait speed were not controlled for and may have influenced the observed biomechanical parameters.

## Conclusion

This study investigated musculoskeletal-derived biomechanical loading patterns in patients with union and nonunion following tibial shaft fractures. The findings indicate that biomechanical loading characteristics are associated with fracture-healing outcomes and evolve over the course of healing. Throughout the healing period, patients who developed nonunion exhibited greater asymmetries between the fractured and contralateral limbs in push-off moment, joint reaction forces, and plantar flexor muscle forces than patients with union. These findings provide a basis for larger longitudinal studies to determine whether biomechanical loading patterns offer additional diagnostic or prognostic value and could facilitate the development of individualized rehabilitation and weight-bearing recommendations.

## Data Availability

The datasets used and/or analysed during the current study are available from the corresponding author on reasonable request.
